# Graphene-Based Catalysts for Ozone Processes to Decontaminate Water

**DOI:** 10.3390/molecules24193438

**Published:** 2019-09-22

**Authors:** Fernando J. Beltrán, Pedro M. Álvarez, Olga Gimeno

**Affiliations:** Departamento de Ingeniería Química y Química Física, Instituto Universitario de Investigación del Agua, Cambio Climático y Sostenibilidad. Universidad de Extremadura, 06006 Badajoz, Spain; pmalvare@unex.es (P.M.Á.); Ogimeno@unex.es (O.G.)

**Keywords:** graphene oxide, ozone, catalytic ozonation, photocatalytic ozonation, water contaminants

## Abstract

The use of graphene-based materials as catalysts in both ozone and ozone/radiation processes is creating interest among researchers devoted to the study of advanced oxidation processes (AOPs) for the degradation of organic pollutants in water. In this review, detailed explanations of catalytic and photocatalytic ozonation processes mediated by graphene-based materials are presented, focusing on aspects related to the preparation and characterization of catalysts, the nature of the water pollutants treated, the type of reactors and radiation sources applied, the influence of the main operating variables, catalyst activity and stability, and kinetics and mechanisms.

## 1. Introduction

Treatment and reuse of wastewater are nowadays key challenging issues facing our planet because of the scarcity of water and the presence of potentially hazardous contaminants that are not safely removed in conventional wastewater treatment plants (WWTPs). Clear examples of this are pharmaceutical compounds or personal care products that for more than two decades have been detected in treated domestic wastewater [1,2,3,4,5,6,7]. Efficient tertiary treatment methods need to be implemented in WWTPs for complete removal of these and other emerging contaminants. Of the tertiary treatment technologies available, only advanced oxidation processes (AOPs) can completely eliminate these pollutants, in contrast to adsorption or membrane processes that transfer the contaminant from the water phase to the adsorbent or the membrane surface. AOPs typically generate hydroxyl radicals (HO·), which are short-life oxidizing species of unselective and fast reactivity towards most of the organic compounds present in water. Rate constants of these reactions are mostly in the range 10^7^–10^10^ M^−1^s^−1^ [8]. 

Ozone processes are AOPs of special interest because of the high oxidizing power of ozone and its ability to produce synergies when combined with other substances and/or agents (other oxidants, catalysts, and/or radiation) [9]. Today, ozone can be considered as a classical oxidant due to its many possibilities for use in water treatment [10]. For instance, ozone is currently applied in water and wastewater treatment plants to improve coagulation, biological oxidation, and disinfection, or simply to remove bad odor and color [11,12,13,14]. The high reactivity of ozone is due to two differentiated mechanisms of reactions. First, ozone can directly react with organic and inorganic species owing to specific moieties (e.g., double carbon bonds or nucleophilic points), and secondly, ozone can also decompose in water, giving rise to oxidizing free radical oxygen species (ROS), which in turn can react with water contaminants. In this second or indirect ozonation pathway, hydroxyl radicals (HO·) are the predominant ROS [9]. This ozonation mechanism depends greatly on pH and the presence of other agents (i.e., hydrogen peroxide, persulfate, catalysts, radiation, or electric current). The first reported ozone AOPs were combinations of ozone with hydrogen peroxide and UV-C radiation [15]. Subsequently, other AOPs systems such as ozone sonolysis, electrochemical ozonation, and catalytic ozonation have become the subject of numerous research works [16,17,18]. In particular, catalytic ozonation has attracted great interest in the world of water research. Its origin dates back to the middle of the twentieth century when ozone decomposition in water was studied in the presence of cobalt ions [19,20]. More studies on homogeneous catalytic ozonation (i.e., with the catalyst dissolved in water) then followed [21]. However, difficulties in separating the catalyst from the treated water constitute a serious drawback of homogeneous catalytic ozonation. As a consequence, heterogeneous catalytic ozonation processes, where a solid catalyst is used to activate the decomposition of ozone and/or improve the adsorption and surface reactions of organics and ozone, are more promising [22,23]. A number of solids have been tested so far as heterogeneous catalysts for ozone reactions in water. These can be broadly classified into the following categories: metal, metal oxides, carbonaceous materials, and complex metal composites [24,25]. Regarding specifically carbonaceous materials, activated carbon both in powder and granular forms, single or multiwalled carbon nanotubes (SWCNT, MWCNT), fullerenes, carbon fibers, carbon xerogels, and, more recently, graphene-based materials, have been successfully used for the catalytic ozonation of water pollutants [26,27,28]. 

Photocatalytic oxidation is an AOP dating back to 1972 when Fujishima and Onda [29] discovered its potential use for water splitting. In this process, a radiation of sufficiently high energy causing the jump of electrons from the valence band to the conduction band of the semiconductor is used. In this way, oxidizing holes (h^+^) created on the valence band are able to oxidize adsorbed hydroxyl ions and generate hydroxyl radicals. A major limitation of some photocatalytic oxidation processes in degrading water contaminants is the inefficiency caused by the recombination of hole and electrons, thus inhibiting the generation of ROS. On account of this, an oxidant, usually oxygen, is used to trap electrons from the conduction band. If ozone is used in addition to oxygen, the process is called photocatalytic ozonation [30]. When trapping electrons, ozone transforms itself into the ozonide ion radical (O_3_^−^), which eventually yields other ROS, mainly hydroxyl radicals, giving rise to an additional means of HO· generation [31]. As a result, photocatalytic ozonation usually has greater oxidizing power than either photocatalytic oxidation (oxygen as electron scavenger) or single ozonation.

The main semiconductor catalyst used thus far in the photocatalytic oxidation of water contaminants is titanium dioxide (TiO_2_) in powder form, particularly that known as P25 [32]. The use of titanium dioxide, however, presents advantages and disadvantages. The former includes stability, activity, and low cost, while a disadvantage is the high band gap (3.2 eV) that makes TiO_2_ inactive under visible light. Another drawback is the particle size of TiO_2_ P25 (about 30–50 nm), which makes the catalyst difficult to separate from water after treatment and is a form of secondary pollution. This is likely the main reason why photocatalytic oxidation has not yet been applied in practice at the full scale. These two disadvantages have given rise to many studies dealing with the preparation of TiO_2_ composites where a non-metal (N, S), metal, metal oxide, or carbon materials are linked to TiO_2_ [33,34,35]. The aim is to reduce the band gap and make TiO_2_ active under visible light and/or to confer properties (e.g., magnetic separability) that facilitate catalyst separation [36]. Another way to overcome the separation drawback is to attach TiO_2_ particles to support materials [37,38,39,40]. Composites made up of graphene-based materials and TiO_2_ have recently been explored as catalysts in photocatalytic ozonation.

**Figure 1:**
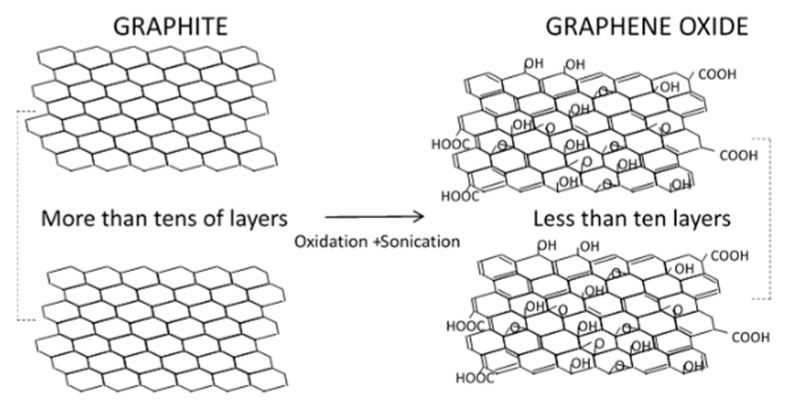
Structures of graphite (with more than ten layers) and graphene oxide (with fewer than ten layers).

Graphene, discovered in 2004, is known as the first synthesized 2D monolayer material constituted by a honeycomb structure of sp^2^ carbon atoms. The structure is actually like that of graphite but with fewer than ten layers (see Figure 1). This structure gives graphene its high electrical conductivity, optical properties, large surface area, mechanical strength, and flexibility [41]. These properties are responsible for the enhancement of adsorption and ozone oxidation of water contaminants as reviewed in this paper. Graphene oxide (GO) and reduced graphene oxide (rGO) are the graphene-based materials most frequently used in ozone catalytic processes. GO is usually prepared by oxidizing graphite [42] to first yield graphite oxide where a fraction of initial sp^2^ carbon atoms change to sp^3^ hybridation by incorporating some oxygen functional groups (hydroxyl, carbonyl, epoxide in the basal plane, and carboxyl at the edges). Graphite oxide is next exfoliated under ultrasonic radiation to obtain GO. In many instances GO is reduced chemically or thermally to obtain rGO, the material most used so far as catalyst in ozone processes [43,44]. In addition to GO and rGO, some graphene-based hybrid materials have been successfully tested as catalysts or photocatalysts in ozonation reactions [45,46,47,48,49,50,51,52,53,54]. The presence of graphene derivatives provides the composite with a higher surface area and, in photocatalytic reactions, suppresses to some extent the electron-hole recombination and/or reduces the band gap of the composite so that it becomes active under visible light.

In this paper, a review of the works published on graphene-based catalysts for both catalytic and photocatalytic ozonation is presented. Reviews on catalytic and photocatalytic ozonation with catalysts other than graphene-based materials and on GO-mediated photocatalysis in the absence of ozone have already been published [30,55,56,57]. However, to the best of our knowledge, there is a gap in the literature regarding ozone processes catalyzed by graphene-based materials.

## 2. Synthesis of Graphene-Based Catalysts for Ozonation Processes 

As mentioned above, GO and rGO are the metal-free graphene-based materials most used in catalytic ozonation processes [58,59,60,61]. The most widely applied method to prepare GO from graphite is based on the Hummers method (i.e., strong oxidation of graphite with KMnO_4_ in sulfuric acid) [62], usually with some improvements to avoid the release of toxic gases [63]. Typically, commercial graphite or another graphite-containing material (e.g., spent lithium-ion batteries [60]) is mixed with concentrated H_2_SO_4_ (95% wt.) in an ice bath. Under vigorous stirring, KMnO_4_ is slowly added to the mixture. The reaction system is kept under stirring until a dark brown solution is obtained. Then, an aqueous solution of hydrogen peroxide (30% wt.) is slowly added to yield a yellow colored aqueous suspension of GO particles. This suspension is filtered and then washed, first with diluted HCl aqueous solution (1:10) to remove metal ions, then with deionized water until the supernatant reaches neutral pH. Finally, exfoliated GO flakes are obtained by ultrasonic dispersion of a GO suspension and removal by centrifugation of the non-oxidized graphitic particles. The degree of oxidation of graphene is reported to have an impact on catalyst performance in ozonation reactions in water. Thus, Ahn et al. [64] prepared graphene materials with different levels of oxidation (namely non-oxidized graphene (nOG), graphene oxide (GO) and over-oxidized graphene oxide (oGO)). Non-oxidized graphene was obtained by treating graphite with (NH_4_)_2_SO_4_ in sulfuric media to expand the structure of graphite. The solid was then recovered, washed, and subjected to physical exfoliation by thermal treatment at 600 °C. Over-oxidized graphene oxide was synthesized similarly to GO but under stronger oxidation conditions. It was observed that the catalytic performance of oGO to decompose aqueous ozone into hydroxyl radicals was much better than GO and nGO. 

Synthesis of rGO using GO as precursor can be accomplished by different reduction methods, including thermal, microwave, chemical, photochemical, photocatalytic, and solvothermal methods [65]. During the reduction process, most of the oxygenated functional groups of GO are removed, thus restoring a large part of the graphitic area, though some oxygen and nitrogen functionalities as well as some defects remain [66]. For its use in catalytic ozonation processes, rGO has been synthesized mainly by thermal reduction under nitrogen or static air atmosphere at 350–700 °C [46,49,59,67], although microwave, hydrothermal, and chemical methods have also been used recently [61,68]. 

In order to improve the catalytic properties of graphene, the designed incorporation of heteroatoms into the carbon framework has been reported [69]. In this sense, rGO has been doped with nitrogen, phosphorous, sulfur, and boron aimed to enhance its catalytic activity in ozone reactions [49,67,70,71]. In all of these works, the preparation of doped rGO was accomplished by thermal treatment of a mixture of GO and the dopant agent. For this purpose, melanine or ammonium nitrate (N-doped), phosphoric acid and ammonium phosphate (P-doped), boric acid (B-doped), and mercaptoacetic acid or benzyl disulphide (S-doped) were the chemicals used. Thermal treatment was typically carried out under nitrogen atmosphere at varying temperatures (160–900 °C) depending on the doping agent.

In addition to bare and doped GO and rGO, some graphene-based nanocomposites have proved useful for catalytic ozonation of water pollutants. These include α-MnO_2_-rGO [45], γ-MnO_2_-rGO [46], and Fe_3_O_4_-GO, TiO_2_-GO and Fe_3_O_4_-TiO_2_-GO [47], obtained by hydrothermal methods. Typically, the preparation of the catalyst is carried out by mixing GO and salts used as the precursor of the metal oxide nanoparticles and transferring the mixture to an autoclave with a Teflon liner. A thermal treatment is then applied to obtain the nanocomposite. The solid sample is finally washed thoroughly and dried in vacuum at room temperature or in an oven at moderate temperature (60–80 °C). MnFe_2_O_4_-rGO nanofibers were also prepared by an electrospinning technique and favorably used in the catalytic ozonation of di-*n*-butyl phthalate [48]. In this case, a viscous homogeneous solution of GO, iron and manganese salts, and polyvinyl pyrrolidone was prepared for electrospinning, applying a voltage of 30 kV. The composite nanofibers were dried and calcined at various temperatures in the 300–600 °C range.

As far as composites used in photocatalytic ozonation studies are concerned, TiO_2_-GO and TiO_2_-rGO are the most frequently used catalysts. Synthesis of these materials has been so far accomplished through liquid phase deposition, hydrothermal, sol-gel, and electrophoretic methods. In the liquid phase deposition method, a dispersion of GO or rGO was treated with (NH_4_)_2_TiF_6_ in a boric acid medium at 60 °C with agitation. After filtration, washing, and vacuum drying, the resulting solid was heated in an oven at 200–350 °C under N_2_ atmosphere, thus obtaining a TiO_2_-GO or TiO_2_-rGO composite, respectively [49]. In the hydrothermal method, GO was treated with a titanium salt or titania in a Teflon-lined autoclave at 120–140 °C. After centrifugation and washing with aqueous HCl and deionized water, the resulting solid was dried in an oven at 80 °C [50]. An electrophoretic method was used to coat a titan grid sheet with N-TiO_2_ and GO [53]. A suspension of N-TiO_2_, GO, and magnesium nitrate in 2-propanol was sonicated and placed in an electrochemical cell between the titan grid sheet and a stainless steel plate. The suspension was subjected to a potential difference of 40 V and the prepared nanocomposite was finally dried in air and calcined at 500 °C. The sol-gel method has been recently used for the purpose of preparing a TiO_2_-GO [54]. In this method, a volume of titanium (IV) butoxide is diluted in isopropanol and distilled water at pH 2 under vigorous stirring. A titania sol is obtained after refluxing this solution and removing the excess of alcohol by heating at 80 °C under vacuum. Then, a volume of GO suspension is added to the titania sol and sonicated for 1 h. After evaporation to dryness under vacuum at 80 °C the solution is heated overnight in an oven at 100 °C.

## 3. Methods Used for The Characterization of Graphene-Based Catalysts

To characterize GO, rGO, and their functionalized forms a number of complementary techniques have been used including the following: scanning electron microscopy (SEM), transmission electron microscopy (TEM), field emission scanning microscopy (FE-SEM), high resolution transmission electron microscopy (HR-TEM), atomic force microscopy (AFM), X-ray diffraction (XRD), adsorption-desorption methods, thermal techniques such as thermal analysis (thermogravimetry (TG), differential thermogravimetry (DTG), differential thermal analysis (DTA)) and temperature-programmed desorption (TPD), Fourier-transform infrared (FTIR), and Raman and X-ray photoelectron (XPS) spectroscopies [72].

The morphology and size of graphene-based materials used in catalytic ozonation have been assessed mainly by straightforward electron microscopy techniques including both SEM and TEM. These methods allow to distinguish between the silk-like structure of GO, the exfoliated rGO, and the multilayer structure of nOG [60,64,73]. Furthermore, the number and thickness of layers in exfoliated structures have been determined by AFM in some instances [61,73]. Also, alterations of the morphology of graphene sheets after doping rGO with N, P, B, or S have been studied with electron microscopy techniques [68]. Regarding graphene-based composites used in catalytic and photocatalytic ozonation of water pollutants, different structures have been revealed by SEM and the higher-resolution TEM, as discussed in Section 7 and Section 8. 

XRD is commonly used to identify bulk crystalline components of solid catalysts. Accordingly, XRD patterns of graphite GO and rGO are quite well established. Graphite shows a peak at 2θ = 26.8° with an interlayer spacing of 0.34 nm, while GO presents a sharp diffraction peak at 2θ = 10.6° corresponding to a d-spacing of about 0.74 nm. After reduction, a diffraction peak of rGO appears at about 2θ = 24.6° with a d-spacing of about 0.38 nm [69]. The XRD technique has also been useful to study the crystal structure of graphene-based composite materials of interest in catalytic and photocatalytic ozonation of water contaminants such as g-C_3_N_4_-rGO [51], MnO_2_-rGO [46], Fe_3_O_4_-GO, TiO_2_-GO, and Fe_3_O_4_-TiO_2_-GO [47], MnFe_2_O_4_-rGO [48], and TiO_2_-rGO [52] among others.

The most common method used to characterize the porous structure of solid catalysts is the adsorption-desorption isotherm of a gas, typically nitrogen at 77 K. From the isotherm a number of parameters can be obtained related to pore surface area and volume, which can provide a fairly complete picture of the pore structure. Typically, GO and rGO show N_2_ isotherms matching with type II isotherm of the International Union of Pure and Applied Chemistry (IUPAC) classification, revealing a mesoporous nature, though the surface area of rGO is much larger than that of GO as a result of the porosity created by the reduction process. Thus, for example, Rocha et al. [71] found specific surface areas (S_BET_) of 23 and 263 m^2^g^−1^ for GO and rGO, respectively. Others have reported even a larger surface area for rGO [68]. The porous structure of graphene-based composites used in ozone reactions in water may be quite different from one another depending on the components and their percentages in the composite as well as in the preparation conditions. 

The surface of GO and, to a lesser extent rGO, contains a number of surface oxygen groups (SOGs) which may play a role in ozone reactions. The TPD technique can be used to ascertain the nature and quantity of these groups from the amounts of CO_2_ and CO released upon heating the sample at controlled conditions [71]. Complementary to TPD methods, even though no FTIR signal can be collected for pristine graphene, this spectroscopy method is frequently used to identify SOG and other functionalities created on graphene-based catalysts used for ozone reactions [48,49,50,52,53,58,68,73]. TG, DTG, and DTA analyses have also been carried out by some researchers to investigate the chemical composition (i.e., carbon content) and thermal stability of graphene-based composites [46,49]).

Raman spectroscopy is a very effective method to characterize the detailed bonding structure of carbon nanomaterials, hence it is considered as the standard tool to characterize graphene-based catalysts. Raman spectra of graphene materials display two broad peaks centered at about 1590 cm^−1^ (G peak) and 1350 cm^−1^ (D peak). The G band is related to the in-plane vibrations of sp^2^ carbon atoms in a graphitic 2D hexagonal lattice, while the D band results from out-of-plane vibrations attributed to defects and disorder in the graphene layer and at the edge of this layer [64]. Therefore, the ratio between the intensities of these Raman signals (D/G band intensity ratio, I_D_/I_G_) is widely used to estimate the density of structural defects in graphitic materials. This ratio increases in GO with respect to graphite due to the increase in disordered structure resulting from the oxygen functionalities produced during the oxidation of graphite. Upon reduction (i.e., rGO), removal of SOG takes place, which repairs defects by restoring the aromatic structure of the graphite lattice. Functionalization of rGO with doping agents (e.g., N-doped rGO) usually brings about an increase in the I_D_/I_G_ ratio because of the introduction of topological defects [68].

XPS spectra are obtained by measuring the kinetic energy of electrons emitted by the material surface (<10 nm) when irradiated with an X-ray beam. This gives information about the elemental composition of the surface of catalysts and the binding states of each element, which is crucial for catalyst research. Most of the reviewed works on graphene-based catalyst for ozone reactions in water make use of XPS, especially when the graphene material is doped or used in a nanostructure with other components. Thus, XPS survey spectra have been used to confirm the presence of foreign atoms on the graphene (doped graphene) and element spectra provide information about the surface functional group, which may be involved in the catalytic mechanism [46,47,48,49,53,59,60,61,64,68,70,71,72,73].

In photocatalysts, UV-Vis diffuse reflectance (DRS) and photoluminescence (PL) spectroscopies are commonly employed as catalyst characterization methods in addition to the techniques presented above. Some DRS and PL results of graphene-based photocatalysts are discussed in Section 8.

## 4. Role of Ozone in Preparing Graphene-Based Catalysts

Due to its high oxidizing character, ozone has also been used to synthesize GO. In fact, it is well known that ozone reacts with carbon materials to form different surface oxygen groups and that treatment at elevated temperature may change their crystallinity and porosity [74,75]. So far, some works have been published about the ozone oxidation of graphite, graphene, and graphene oxide. Krawczyk [76] reported the ozonation of an expanded graphite bed at ambient temperature and at 140 °C. After ozone treatment, increases of porosity and surface area were observed together with changes in the morphology of the material, especially at 140 °C. The results confirmed the formation of surface defects as well as edges that can be considered as active centers of graphitic materials. New distributions of C–O and C = O groups were observed after ozone treatment, as revealed by XPS. An appreciable increase in the concentration of surface oxygen groups is reported to occur at room temperature due to formation of C–O bonds. At high temperature, however, an increase in the BET surface area occurred simultaneously with a reduction of the surface oxygen concentration. The ozonized expanded graphite has been applied in the electrochemical oxidation of phenol showing higher degradation efficiency than non-treated expanded graphite. Baldissarelli et al. [77] synthesized GO (or expanded exfoliated graphite oxide, as they named it) by ozonation of a powdered expanded graphite oxide during 14 h, (1 L min^−1^ gas flow rate, ozone concentration not given) followed by 1 h sonication. They subsequently used TiO_2_ P25 to prepare TiO_2_-GO composites by hydrothermal and reflux methods. The photocatalytic activity of these composites for the oxidation of methylene blue (MB) in air using a 150 W TQ UV lamp emitting radiations between 200 and 280 nm wavelength was evaluated. They compared their results with those obtained with TiO_2_ P25 and TiO_2_-GO composite prepared by the classical Hummers method (HTiO_2_-GO). The relatively low percentage of GO in the composites (4.97%) made it so the specific surface areas of both TiO_2_-GO composites were close to that of TiO_2_ P25 (about 50 m^2^ g^−1^). The prepared TiO_2_-GO composites absorbed visible light and their band gaps were similar to each other (about 3.13 eV for TiO_2_-GO from ozonation and 3.10 eV for HTiO_2_-GO). Also, formation of Ti–O–C bonds was confirmed since the mixture of GO+TiO_2_ did not show any absorption in the visible region. However, no XPS data were given to support the presence of Ti-O-C in the TiO_2_-GO composite. An almost two-fold increase of the D/G band intensity ratio (I_D_/I_G_) of the GO prepared by ozonation compared to the starting graphite was measured. This indicates an increase of sp^3^ carbons and, hence, an increase of oxygen containing groups in GO. Finally, when checking photocatalyst activity the authors found apparent pseudo-first order rate constants of methylene blue removal of 0.102, 0.125, 0.342, 0.270, and 0.178 min^−1^ for direct UV photolysis, TiO_2_ P25, TiO_2_-GO (ozone+thermal method of preparation of GO), TiO_2_-GO (ozone+reflux method of preparation of GO), and HTiO_2_-GO, respectively. Accordingly, the results showed better performance of the composites prepared by graphite ozonation. Zhang et al. [78] also used ozone as oxidant to form GO from graphite. In this work, concentrated ozone (20–30%) was applied to powdered graphite, previously treated with NH_4_NO_3_. From TEM images the authors observed ordered graphitic lattices on the basal plane and defective zones at the edges of sheets. Fewer than ten layers were identified from atomic force microscopy so that the material could be identified as GO. Raman study revealed formation of sp^3^ C from the increase of the I_D_/I_G_ up to 0.59, higher than that of graphite (0.23). XPS analysis also showed an increase of surface oxygen groups (hydroxyl, carbonyl, and carboxyl) compared to the amounts in graphite. The catalytic activity of the prepared GO was checked in the wet air oxidation of benzyl alcohol to benzaldehyde at 150 °C in the presence of 2,2,6,6-tetramethylpiperidin-1-oxyl as a co-catalyst. The authors reported a significant increase of benzylalcohol conversion and selectivity towards benzaldehyde compared to those observed when GO prepared by the Hummers method was applied. The as-prepared GO catalyst showed fairly good stability and activity after six cycles of reuse.

Studies of graphene ozonation to yield GO have also been reported. Mulyana et al. [79] studied the oxidation-reduction process of graphene with UV/O_3_ and UV, then analyzing the properties of the GO and reduced rGO materials obtained, respectively. The starting graphene was produced by chemical vapor deposition of methane gas. Graphene was then simultaneously treated with ozone and UV radiation of 184.9 and 253.7 nm wavelengths. UV radiation decomposed ozone into radical oxygen atoms that eventually reacted with carbon atoms in graphene to form surface epoxides groups. In a second step, only UV radiation was applied to reduce GO. Ozone photolysis periods lower than 10 min led exclusively to formation of epoxide groups, while increasing the oxidation time up to 15 min led to the appearance of some carbonyl groups as XPS characterization showed. This led to the formation of some lattice defects. During all ozonation time C–C bonds continuously disappeared. This contrasts with XPS results observed in pristine graphene that only showed the peak corresponding to C–C sp^2^ bonds. This process yields homogeneous oxygen epoxide groups on the surface of the graphene layer. In a second step, the GO formed after the ozone application was subjected to 6-min UVC radiation periods. The amount of oxygen in GO was reduced and C–C bonds increased, leading to rGO. This sequence of UV/O_3_ followed by UV radiation was conducted in consecutive cycles with the same results, that is, formation of homogeneous epoxide groups on GO during the ozonation period and their removal during the UV radiation period. The results were confirmed by Raman measurements of I_D_/I_G_ ratio. The authors concluded that the processes studied can provide valuable information about chemical functionalization of GO.

Ozone has also been applied to modified already prepared GO in order to unveil unknown aspects of the role of ozone in GO oxidation or decomposition. For instance, Yang et al. [80] carried out ozone reactions with GO prepared from the Hummers method to introduce oxygen-containing groups, widen the optical band gap, and improve fluorescence. They followed the fluorescence properties of ozonized GO (oz-GO) that they related to the process mechanism. The increase of fluorescence properties was attributed to the presence of new oxygen groups as fluorophores. These results were confirmed from XPS, Raman and optical spectroscopy results. From UV-vis absorption of GO and oz-GO, they observed the typical absorption peak at about 230 nm, assigned to the π−π* transition of aromatic sp^2^ domains. With increasing ozonation time this peak gradually experimented a blue shift, suggesting that the oxygen containing groups reduced the π−π* domains to widen the gap. The fluorescence changes indicated that the ozone first plays a role as oxidant to create oxygen containing groups and later (with prolonged ozonation time) brings about an erosion effect leading to the release of CO and CO_2_ and the creation of new surface. The authors finally concluded that ozone can tune the GO structure in a simple, economical, efficient, and controllable way where ozonation time is the key parameter. In a similar work, Hasan et al. [81] studied the ozonation of a commercial GO to control its band gap. Aqueous suspensions of GO were ozonated inside an ultrasonic bath for periods of up to 35 min in order to alter the optical band gap. The authors also observed the increase of oxygen containing groups (C–O and C = O), an increase in GO fluorescence intensity, and significant (100 nm) blue shifts in emission maxima with ozonation time as well with a decrease of sp^2^ cluster size. As a result of ozonation, band gap increased from 1.96 to 2.56 eV for sp^2^ cluster sizes changing from 1.86 to 1.03 nm, respectively. In summary, two competing processes were confirmed to take place: (1) The addition and rearrangement of functional groups on the GO surface resulting in a blue shift and an increase in the emission intensity and fluorescence quantum yield; and (2) deterioration of GO flakes, leading to fluorescence quenching. 

Finally, the role of ozone as an intermediate formed during the GO synthesis by the well-known Hummers method has been investigated. Recently Chen et al. [82] reported the formation of ozone in a water-addition modified Hummers method to prepare GO sheets with tunable amounts of hydroxyl and epoxide groups without destroying their structural integrity. The authors claimed that in excess of water, exfoliation of oxidized graphite releases ozone, which is formed due to KMnO_4_ reduction in a concentrated H_2_SO_4_ medium as Dzhabiev et al. [83] had previously reported. The presence of ozone as an intermediate was corroborated from UV-visible absorption spectroscopy results that showed the characteristic ozone band at 254 nm (Hartley band). Chen et al. [82] proposed the following mechanism of reactions:

(1) $$3H_{2}O+2\mathrm{Mn}(\mathrm{VII})\overset{}{\to }O_{3}+2\mathrm{Mn}(\mathrm{IV})$$

(2) $$O_{3}\overset{\mathrm{Mn}(\mathrm{IV})}{\to }O_{2}+O$$

(3) $$O_{3}{+H}_{2}O\overset{\mathrm{Mn}(\mathrm{IV})}{\to }O_{2}+2\mathrm{HO}\cdot$$

(4) $$\mathrm{Graphite}\overset{O_{3}/\mathrm{HO}\cdot /O}{\to }\mathrm{Graphite}\;\mathrm{oxide}$$

Groveman et al. [84] tried to complete this mechanism by explaining how reaction (4), the ozonolysis step, develops. These authors also prepared GO following a similar Hummers method with excess water. At their experimental conditions (40 °C and subsequent rise to 60 °C) they favored the formation of GO with a high degree of oxidation and they smelled the typical ozone odor during the process. They performed an extensive study of GO characterization, confirming the presence of numerous species (hydroxyl radical, atomic oxygen, peroxymonosulfate ester, etc). From temperature-programmed desorption coupled with mass spectrometry (TPD-MS) measurements they observed a peak at 225 °C (*m*/*z* 48) compatible with the existence of ozone but also with the presence of SO, so the presence of ozone could not be definitively confirmed. With XPS results they identified the presence of hydroperoxides (C–O–O–H) and persulfates (C–O–O–S) (peaks at 286.2 eV and 289.0 eV, respectively), which were also observed in the O1s spectrum at 533.6 eV and 534.7 eV, respectively. However, definitive proof of the presence of ozone was not found. Then, they studied a possible mechanism with participation of ozonolysis from density functional theory (DFT) calculations. DFT is a modelling method based on computational quantum mechanics used to investigate the electronic or nuclear structure of atoms, molecules and condensed phases, among other groups [85,86]. With DFT calculations based on their experimental findings and Lerf–Klinowski [87] and the Szabo–Dékány [88] GO structural models they could establish a mechanism of reactions (see Figure 4 in [84]) where the existence of primary and secondary ozonides and a Criegge intermediate are key species of GO formation. They finally concluded that their modified Lerf–Klinowsky model was structurally better than the Szabo–Dékány model and accounts for a sterically hindered ozonolysis on progressively oxidized graphite.

## 5. Main Properties of Graphene-Based Catalysts 

From preparation methods, characterization techniques and ozone role in GO formation, the main properties of GO based catalysts can be established and are summed up in this section.

First, it should be reminded that the method of preparation of graphene-based catalysts highly affects their properties. The way of preparation, on the other hand, depends on the ozonation process where the catalyst will be applied. 

For catalytic ozonation, strong oxidation methods are applied followed by exfoliation. The most important property in the prepared catalysts is the presence of different SOG and defect sites in GO layers that can be tuned through the type of oxidation in the synthesis method. With weak to moderate oxidation of graphene, most of the generated SOG are epoxides and some are hydroxyl groups in the basal plane and carboxylic groups at the edges. With strong oxidation (for instance, by applying high amounts of ozone or high ozonation times on graphene), carbonyl groups are introduced which means the appearance of defect sites and increases in catalyst activity. If GO is reduced (for instance by applying UV radiation), the SOG concentration drastically decreases and specific surface area increases. XPS and FTIR are the techniques typically used to identify SOG. Also, Raman spectroscopy through measurements of I_D_/I_G_ ratio allows the estimation of the sp^3^ to sp^2^ carbon atom ratio. This is another way of let one know the degree of oxidation. Another important property is the specific surface area and porosity that can be improved by reducing GO to rGO. Specific surface area and porosity are determined through nitrogen adsorption–desorption isotherms and application of BET and BJH methods. 

Regarding photocatalytic ozonation, the already-prepared GO is linked to an active semiconductor, mainly TiO_2_ through different methods (thermal, reflux, sol-gel). The main property of TiO_2_-GO catalysts is the capacity to absorb visible light. GO presents band gap lower than 3 eV, which makes this material absorb visible light. The presence of GO then makes the TiO_2_-GO composite a visible light-active catalyst. This property is confirmed with the use of the UV-Vis-DRS technique. Distribution of metals, metal oxides, and doping agents on GO hybrid materials is another property to be considered. TEM and SEM are the techniques needed to this task. Finally, XRD is the technique used to examine the degree of crystallinity of graphene-based materials. However, in some instances, due to the low percentage of graphene in photocatalysts, the characteristics peak corresponding to GO (2θ about 10.6°) is not usually detected.

## 6. Target Pollutants Used in Ozonation Catalyzed by Graphene-Based Materials

As shown in Table 1, most water contaminants used as target models in studies of catalytic and photocatalytic ozonation mediated by graphene-based materials belong to three main groups: phenols, pharmaceutical compounds, and low-molecular-weight carboxylic acids. There are also studies on removal of some other types of contaminants such as plasticizers (di-*n*-butyl phthalate (DBP) and bisphenol, though the latter also belongs to the phenol family), the insecticide N, N-diethyl-m-toluamide (DEET), the surfactant perfluorooctanoic acid (PFOA) which is usually found in domestic wastewater, and p-chlorobenzoic acid (p-CBA), which is frequently used as a probe compound to check the hydroxyl radical exposure in ozone processes through the determination of the R_CT_ parameter [89]. 

Phenols (especially phenol itself) are very often used as model compounds because of their presence in many wastewaters [90,91,92]. The direct reactions of these compounds with ozone are very fast, particularly at a pH above 5 [93]. For example, the presence of hydroxyl group in a phenol aromatic ring activates the electrophilic substitution reactions of ozone, the rate of which increases with pH due to the dissociating character of phenols and the stronger activating agent of O^-^ as compared to HO^-^. Ozonation alone is, then, an efficient process to remove phenols from water and the addition of a catalyst and/or light is not really necessary. However, the use of phenols as model compounds in ozone catalytic processes is mainly due to total organic carbon (TOC) removal achieved since it is well known that ozonation alone is not capable of reducing TOC levels more than 30%, especially when target compounds are treated when dissolved in wastewaters. Low molecular weight carboxylic acids show the opposing situation regarding ozone reactivity. These compounds are classified as very refractory to ozone direct reactions; rate constants of these reactions are lower than 1 M^−1^s^−1^ with some exceptions (e.g. formic acid) and are usually found as end products of ozonation processes or advanced oxidation processes [94,95,96]. For example, oxalic acid is often used as a model compound to check the performance of catalysts or advanced oxidation processes because it only reacts with hydroxyl radicals. The refractory nature of oxalic acid is deduced from the value of the rate constant of its reaction with the hydroxyl radical, which is of the order of 10^7^ M^−1^s^−1^ [8], that is, about two to three orders of magnitude lower than the rate constants of the reactions of these free radicals with most organic compounds. Finally, the third important group of compounds treated in catalytic ozone studies are pharmaceuticals (antibiotics, anti-inflammatories, etc.) or compounds used for health purposes such as iopromide X ray contrast media or benzotriazole protein inhibitors, though the latter are also used as an anticorrosion substance, for instance, on copper surfaces. These compounds are identified at ngL^−1^ to μgL^−1^ levels in urban wastewater treatment plant effluents, representing a high potential risk for living beings. A long residence time of antibiotics in water favors resistance of bacteria and genes, which today constitutes a high health risk due to the many illnesses that these pathogens generate. Antibiotics are, then, the main target micropollutants that can be removed from water through ozone catalytic processes. Unlike phenols or low-molecular-weight carboxylic acids, pharmaceuticals cannot be classified as fast or slow ozone-reacting compounds since their direct reaction rate constants vary from less than 0.8 M^−1^s^−1^ for iopromide or 3.1 M^−1^s^−1^ for the primidone–ozone reaction up to 4.15 × 10^5^ M^−1^s^−1^ in the case of the sulfamethoxazole–ozone reaction. This high variability of ozone reactivity is due to the selective character of ozone, which mainly reacts with compounds possessing nucleophilic points in their molecules (double or triple carbon bonds, aromatics with substituting groups that activate the electrophilic substitution reactions such as phenols, etc.).

## 7. Catalytic Ozonation

Heterogeneous catalytic ozonation is recognized as a novel alternative for advanced treatment of wastewater. It has attracted great attention in recent years due to its high capacity for the mineralization of industrial organic pollutants [22,23]. In this sense, metal-based catalysts, especially noble metals and transition metal oxides, have been proved to be catalytically active for ozone decomposition. However, potential secondary contamination from metal leaching can scarcely be avoided due to the strong oxidative capacity of ozone. Looking ahead to a sustainable future, the development of metal-free catalysts with excellent performance for wastewater remediation is in urgent demand. In this respect, graphene-based materials are seen as alternative catalysts for ozone reactions aimed at water treatment. Table 2 shows the research studies carried out so far in which graphene-based materials are used for the catalytic ozonation of water pollutants.

As apparent from Table 2, the catalytic ozonation of water contaminants mediated by graphene-based materials is still an incipient area of research, as fewer than 20 works have been published so far. Nevertheless, new studies in this field are expected, since promising results have been found in the reported works where graphene materials enhanced the adsorption of pollutants and promoted their oxidation through ozone decomposition and generation of ROS. Doping of GO and rGO with N, P, and B or combining them with other nanomaterials (e.g., MnO_2_ particles) has also been proved useful for catalytic purposes. Therefore, the design of graphene-based materials with enhanced catalytic activity towards ozone transformation into ROS by selectively tuning the type and density of surface active sites may become an active area of research. Here a summary of catalytic activity, stability, and reusability of the investigated materials to date is presented. In addition, a brief discussion of kinetic and mechanistic aspects is provided.

### 7.1. Catalytic Activity

To test the activity of graphene-based materials in the catalytic ozonation of water pollutants, two types of experimental approaches are usually carried out. In one of them, ozone decomposition in ROS in the presence of the catalyst but in the absence of water pollutants is explored. Thus, for example, Ahn et al. [64] investigated the decomposition of aqueous ozone in hydroxyl radicals in the presence of several graphene-based materials, including GO, nGO, and oGO. Based on the R_CT_ concept and the HO· yield (i.e., the ratio of produced HO· to consumed O_3_) they found the following order of effectiveness in ozone decomposition at acidic to neutral pH: oGO > GO > nOG > ozone alone. This clearly indicates that the oxidation degree of the graphene material had an effect on the ozone chain decomposition. Similar results were observed by other researchers [73]. In a second, more common approach, one or more target pollutants are selected and their removal rates in the presence and absence of the catalyst are measured and compared. Also, adsorption experiments (i.e., no ozone provided) have been carried out. Catalytic activity is then considered satisfactory if the removal rate of catalytic ozonation is higher than the combined effect of adsorption on the catalysts and ozonation alone. As seen in Table 2, semi-batch ozonation is the preferred operation mode, though some studies have also been performed batchwise.

Adsorption of water pollutants onto graphene-based materials theoretically depends on a number of factors, including the properties of the compound to be adsorbed (e.g., functional groups), the graphene material (e.g., surface area, pH_PZC_), and the aqueous solution (e.g., pH). In general, the works listed in Table 2 report limited adsorption of organic compounds onto graphene-based materials. Thus, for example, Liu et al. [58] observed less than 15% DEET adsorption onto GO, and Yoon et al. [73] found that neither p-chlorobenzoic acid (p-CBA) nor iopromide adsorbed onto oGO, GO, or nOG. Despite having a large surface area (265–300 m^2^g^−1^), a synthesized rGO was not able to adsorb p-CBA to a significant extent [59]. In this respect, negligible adsorption of some low molecular weight carboxylic acids (i.e., oxalic, acetic, and formic acids) as well as acetyl salycilyc acid, p-HBA and 4-nitrophenol onto a prepared rGO (362 m^2^g^−1^) has also been reported [60]. Doping GO and rGO with heteroatoms did not enhance adsorption of organic compounds from aqueous solution, as observed by various researchers [67,68,70,71]. Regarding graphene-based composites, different adsorption capacities have been reported. Thus, for example, Wang et al. [46] observed negligible adsorption of 4-nitrophenol onto γ-MnO_2_-rGO nanoarchitectures, while Ren et al. [48] found about 30% DBP removal by adsorption on MnFe_2_O_4_ and MnFe_2_O_4_ nanofibers. In general, taking into account the low level of adsorption onto graphene-based materials used for the catalytic ozonation of water contaminants, most of the researchers consider that adsorption contributes to a small extent to the removal of water pollutants by catalytic ozonation. Therefore, they consider single ozonation experiments (i.e., absence of catalyst) as a benchmark for examining the catalytic performance of graphene-based materials.

As a rule, graphene-based materials such as GO, rGO, doped rGO, and graphene-containing composites lead to higher levels of contaminant removals than single ozonation, thus suggesting good catalytic activities. For example, Liu et al. [58] observed between 85% and 98% DEET removal after 10 min of an O_3_/GO process (the percentage of DEET removal depended on the dose of GO, ranging between 20 and 100 mg·L^−1^) while about 40% DEET was achieved by single ozonation in the same reaction time. Wang et al [60] reported 95% and 100% oxalic acid removal within 1 h of ozonation at pH 3 using 0.1 g·L^−1^ of rGO obtained from pure graphite and a spent lithium battery (LIB), respectively, while ozone in the absence of any rGO barely removed 5% of oxalic acid. The rGO from LIB also showed extraordinary catalytic ozonation activity towards the removal of acetic acid, formic acid, acetyl salicylic acid, 4-nitrophenol, and p-HBA. A comparison of the activities of one GO and two rGOs prepared at different temperatures to degrade p-HBA showed that though both types of catalysts were active for catalytic ozonation, the degradation and mineralization efficiencies achieved with the rGOs were significantly higher [59].

Some heteroatom doped-graphene materials have shown extraordinary activity in ozone reactions. Thus, for example, Yin et al. [67] found that r-GO improved ozonation removal rate of sulfamethoxazole but to a lesser extent than the N-rGO and P-rGO derivatives. The better performance of doped catalysts could be attributed to the catalytic sites created by atom doping. Similar results were found by Song et al. [70], who employed rGO doped with different heteroatoms (N, P, B, and S), their performances being evaluated in terms of the degradation of refractory organics (p-CBA and benzotriazole) and bromate elimination simultaneously. Doping with heteroatoms except sulfur significantly improved catalytic ozonation activity of rGO, the catalytic activity ranking being as follows: N-rGO > P-rGO > B-rGO > rGO > S-rGO. Other works have also shown superior catalytic activity of N-doped rGO for the degradation of 4-nitrophenol [68] and oxalic acid and phenol [71].

Composites containing r-GO have also been shown to be active towards the degradation of water pollutants by ozone. Li et al. [45] synthesized an α-MnO_2_-rGO nanostructure by a hydrothermal method and used it as catalyst for semi-batch ozonation of bisphenol A (BPA). Catalytic activity of α-MnO_2_-rGO towards BPA removal by ozonation was compared to that of bare rGO and α-MnO_2_ particles. While r-GO showed no appreciable catalytic activity, α-MnO_2_ and α-MnO_2_-rGO increased BPA removal percentage from ca. 20% (single ozonation) to ca. 70% and 95%, respectively (see reaction conditions in Table 2). Accordingly, apparent first-order rate constants were successfully calculated to be 3.52 × 10^−3^ min^−1^ (O_3_), 3.19 × 10^−3^ min^−1^ (O_3_/rGO), 1.98 × 10^−2^ min^−1^ (O_3_/α-MnO_2_) and 3.92 × 10^−2^ min^−1^ (O_3_/α-MnO_2_-rGO). This means that the presence of the α-MnO_2_-rGO catalyst increased by 11-fold the apparent rate constant for BPA degradation by ozone. This improved catalytic efficiency of α-MnO_2_-rGO with respect to α-MnO_2_ was attributed to the larger surface area (102 and 59 m^2^g^−1^, respectively) and the effect of r-GO on the acceleration of electron transfer processes. A γ-MnO_2_/rGO composite also proved to have much greater catalytic activity towards the degradation of 4-nitrophenol than individual rGO and MnO_2_ particles [46]. Complete removal of 4-nitrophenol was achieved in about 60 min by single ozonation, ozonation in the presence of rGO, and MnO_2_-catalyzed ozonation. In γ-MnO_2_-rGO-mediated ozonation, 100% 4-nitrophenol removal was attained in about 30 min. Moreover, 4-nitrophenol mineralization achieved after 1 h of ozonation was 16%, 20%, 50% and 80% for O_3_, O_3_/r-GO, O_3_/MnO_2_ and O_3_/γ-MnO_2_-rGO systems, respectively. Accordingly, the γ-MnO_2_-rGO hybrid catalyst showed higher catalytic activity than the traditional MnO_2_ catalyst. Ren et al. [48] fabricated α-MnFe_2_O_4_-rGO nanofibers with catalytic activity in the ozonation of DBP. Under the conditions used in the experiments, the hybrid catalyst containing 5% rGO exhibited the best performance in the removal of DBP, with about 80% removal in 1 h (ozone alone barely removed 30% DBP in that time). In addition to rGO, GO has also been used to prepare nanocomposites with catalytic ozonation activity. In this line, Jothinathan and Hu [47] synthesized Fe_3_O_4_-GO, TiO_2_-GO, and Fe_3_O_4_-TiO_2_-GO nanoparticles and demonstrated their ability to decompose ozone into HO·. Thus, R_CT_ values of Fe_3_O_4_-GO, TiO_2_-GO, and Fe_3_O_4_-TiO_2_-GO catalysts at a dose of 20 mg·L^−1^ were about 2.97 × 10^−9^, 1.77 × 10^−9^ and 3.01 × 10^−9^, respectively, while those corresponding to single ozone and the peroxone process (O_3_/H_2_O_2_) were 10^−10^ and 3.83 × 10^−9^, respectively.

### 7.2. Stability and Reusability

Despite their relatively good activity, a possible drawback of graphene-based materials in ozone reactions is related to the attack of ozone on the carbon framework. Thus, for example, Song et al., [61], using a GO for the degradation of p-CBA and benzotriazole, observed an increase of TOC during the process which was likely a result of the attack of ozone and/or ROS formed onto the GO structure. Also, previous studies have suggested that graphene-based materials used in catalytic oxidation of water pollutants presented poor stability due to both changes in the surface chemistry and coverage of active sites by reaction intermediates [115]. Therefore, stability and reusability studies are crucial when testing these catalysts.

Several authors from Table 2 examined catalytic stability and reusability of graphene-based catalysts through multiple runs. Typically, the catalyst was recovered after each run by vacuum filtration, thoroughly washed with ultrapure water, and dried overnight before use in a new run. In addition, parallel experiments were carried out for each run to compensate for the loss of catalyst during the withdrawing and washing processes. Catalytic activity was measured in each run and a sample of catalyst was usually characterized after use and compared with the fresh catalyst [59,60,68]. 

Wang et al. [59] reused a sample of rGO up to five times for the ozonation of p-HBA, and noticed a considerable loss of catalytic activity with the repeated uses. As the catalyst was successfully regenerated by thermal treatment, the authors concluded that the loss of catalytic activity could be attributed to changes in the surface chemistry and adsorption of degradation intermediates rather than to weight loss in the recovery process. The researchers examined the reused rGO by N_2_ adsorption and XPS and observed dramatic changes in textural properties (i.e., BET surface loss) and the nature of SOG on the rGO surface, with a significant loss of hydroxyl and carboxyl groups which were transformed into carboxyl groups. Similar results were observed for a rGO obtained from spent LIB [60]. In this case, the rGO was utilized in a four-cycle successive test degrading 4-nitrophenol. Loss of catalytic activity was evident from the decrease in the pollutant and TOC removal rates. Thus, percentage of TOC removal in one-hour experiments decreased from 92% with the fresh rGO to 62% with the four-time reused catalyst.

Given that N-rGO showed the best catalytic ozonation performance, doped rGO was used to study the stability and reusability of these graphene materials [67,68,70] by successive three-, four-, or five-run catalytic tests. Wang et al. [68] found that a slight deactivation of the catalyst was produced; through XPS and N_2_-sorption studies, these researchers concluded that compared with surface chemistry changes, variation in physical properties played a crucial role for catalyst deactivation. Yin et al. [67], however, claimed that doped rGO had good stability (i.e., stable layered structure and surface chemistry) and that it can be reused for the long-term run after a thermal annealing treatment. Song et al. [70] observed that N-rGOs were more stable and reusable than other N-doped graphene-based materials.

Some graphene-based composites used in the catalytic ozonation of water pollutants have also been the subject of stability studies [45,46,48]. Interestingly, Li et al. [45] found that the hybrid material α-MnO_2_-rGO showed more stability than commercial MnO_2_ particles in catalytic ozonation of bisphenol A. After five consecutive runs, degradation of bisphenol A decreased by about 10% with α-MnO_2_-rGO nanoarchitectures and around 40% with bare MnO_2_ particles. Deactivation of α-MnO_2_-rGO was mainly attributed to changes in the Mn oxidation state (from Mn(III) to Mn(IV)) upon ozonation as detected by XPS spectra of Mn 2p region. For a γ-MnO_2_-rGO hybrid catalyst the authors also claimed good stability without Mn leaching (<0.5 mg·L^−1^), though some efficiency loss was observed when it was repeatedly used for the ozonation of 4-nitrophenol. Strong adsorption of reaction intermediates was pointed out as the main cause of this minor catalytic activity loss [46]. Similar conclusions were drawn by Ren et al. [48] who observed a small depression of catalytic activity of an α-MnO_2_-rGO nanofiber after four consecutive runs of DBP ozonation. In this case, DBP removal in one-hour runs decreased from 87.2% (fresh catalyst) to 83.2% (catalyst reused four times).

### 7.3. Kinetic and Mechanism Studies

Taking into account the excellent activities observed for rGO, doped rGO, and rGO-based composite materials, some authors from Table 2 further investigated reaction kinetics through a pseudo-first order reaction model [45,46,58,59,60,67]. Based on the rate constants obtained, Yin et al. [67] found that N-rGO and P-rGO enhanced sulfamethoxazole degradation by ozone by a factor higher than 2.5 with respect to ozonation alone. Liu et al. [58] studied degradation of DEET using O_3_/GO, and obtained a pseudo-first-order rate constant that was almost six times higher than that of single ozonation. Similar results were achieved by Wang et al. [59,60] using rGO for the degradation of 4-nitrophenol and p-HBA. Regarding graphene hybrid materials, much higher first-order rate constants have also been reported for catalytic ozonation compared to single ozonation [45,46].

The presence of graphene oxide materials significantly enhances decomposition of ozone molecules to generate ROS such as hydroxyl radical (HO·), superoxide radical (·O_2_¯) and singlet oxygen (^1^O_2_) for eliminating organic pollutants. However, the dominant ROS during the catalytic ozonation process is still subject to debate [59]. Some researchers observed the effect of the presence of different scavengers by conducting radical scavenging tests or liquid phase electron spin resonance (ESR) in order to clarify a possible mechanism of catalytic ozonation by heteroatom-doped/reduced graphene oxides. For instance, Wang et al. [68] used tert-butanol (tBA) to scavenge hydroxyl radicals with p-benzoquinone (p-BQ) as the radical scavenger for^.^O_2_¯ and sodium azide (NaN_3_) for quenching of ^1^O_2_. 5,5-Dimethyl-1-Pyrroline-N-Oxide (DMPO) was employed as the spin trapping agent for capturing HO· and ·O_2_¯ with characteristic signals under ESR. It was found that ·O_2_¯, HO· and ^1^O_2_ were generated by catalytic decomposing of ozone molecules and possibly for 4-NP degradation [68]. Liu et al. [58] also used tBA to evaluate the contribution of hydroxyl radical to the oxidation of DEET by catalytic ozonation using GO. Again, Wang et al. [59] used ESR and radical competition tests which revealed that superoxide ion radical (·O_2_¯) and singlet oxygen (^1^O_2_) were the reactive oxygen species (ROS) for p-HBA degradation. 

On the other hand, only a few works related to identification of intermediates in catalytic ozonation by reduced (and doped) graphene oxide materials have been found. Wang et al. [68] investigated the intermediates in catalytic ozonation of 4-NP by time of flight mass spectroscopy. Mass spectroscopy (MS) with electrospray ionization (ESI) in a negative mode was utilized for detecting reaction intermediates and revealing the mineralization process. MS results revealed that p-HBA was oxidized to produce small molecular carboxylic acids [59]. 

Some researchers have proposed mechanisms for catalytic ozonation by heteroatom-doped/reduced graphene oxides. For instance, Song et al. [70] claimed that surface oxygen-containing functional groups and π electrons in the carbon layer structure promoted ozone decomposition to form ^.^O_2_¯ and H_2_O_2_. The formed ROS led to complete refractory organic compound degradation and bromate elimination. Song et al. [70] also identified main roles of chemical functional groups, doped atoms, free electrons, and delocalized π electrons, as well as the contributions of these active centers to the formation of ROS such as hydroxyl radicals, superoxide radicals, singlet oxygen, and H_2_O_2_. A very complete mechanism of doped graphene catalytic ozonation is also reported in this work for the treatment of p-CBA and benzotriazole (BZA). 

Finally, some works have made use of the DFT to study aspects related to the mechanism of graphene-based catalytic ozonation. As mentioned above, Groveman et al. [84] dedicated their study to elucidate the role of ozone in the formation of GO. Wang et al. [60] studied the catalytic ozonation (GO as catalyst) of six compounds; three of them were low-molecular-weight saturated carboxylic acids (oxalic, formic, and acetic acids) and the other three were aromatics (acetylsalicylic acid (ASA), p-HBA, and p-nitrophenol (p-NP)). They prepared GO materials from spent graphite anodes used in lithium batteries and identified different defective sites on the catalyst by XPS, Raman spectroscopy, and other techniques. DFT was applied to study the mechanism of ozone adsorption on different defective sites of the GO layers. The authors concluded that defective sites and to a lesser extent SOG were the dominant active sites for ozone decomposition. They also observed that the presence and concentration of ROS varied upon the structure of organic pollutants. Thus, from radical scavenging tests and EPR results, the hydroxyl radical was identified as the dominant ROS for carboxylic acid degradation, while ozone, superoxide ion radicals, and singlet oxygen were responsible for the destruction of the phenolic pollutants. Yin et al [67] reported the degradation of the antibiotic sulfamethoxazole (SMX) by ozone catalyzed with GO. Non-doped and N (N-GO)- or P (P-GO)-doped GO were synthesized in this work. The authors applied DFT to study the mechanism of SMX degradation by simulating the geometry optimization of the antibiotic and defining Fukui functions to describe the activity of orbital-controlled reactions [116,117]. The larger value of this function indicated the higher reactivity of the corresponding site. It was suggested that sulfur atom was a site susceptible to the attack of oxidative species, resulting in the cleavage of S–N and S–C bonds adjacent to the S atom. The DFT results were in good agreement with the proposed degradation pathways based on GC-MS spectroscopy results.

## 8. Photocatalytic Ozonation

Only eight works on graphene-based photocatalytic ozonation have been published so far (see Table 3). Together with the work on catalytic ozonation, this means that graphene-mediated ozone processes (especially photocatalytic ozonation) are emerging ozone-AOPs which are starting to be investigated. In photocatalytic ozonation, the graphene material is, as in catalytic ozonation, the corresponding oxide (GO) or rGO, depending on the surface oxygen groups. However, one important difference between both ozone processes is that GO or rGO are used in photocatalytic ozonation as dopants of some semiconductor material, especially TiO_2_, and not as the main and only catalyst, as occurs in catalytic ozonation. As is shown in Table 3, in all cases but one the main catalyst is TiO_2_, the exception so far being rGO-doped ZnO.

### 8.1. Specific Catalyst Characterization Techniques

As occurs in catalytic ozonation works, the prepared composite is characterized with the techniques already listed and commented on in Section 3. One of these techniques, however, is particularly specific for photocatalysis. This is ultraviolet-visible diffuse reflectance spectrometry (UV-Vis-DRS), which allows the determination of the wavelength range of radiation that the solid can absorb and the bandgap of the photocatalyst, a key parameter of this oxidizing system. GO and rGO-containing composites show high absorption capacity of visible radiation that contrasts the null or nearly-null visible absorption of ZnO or TiO_2_, as can be observed in the works of Wu et al. and Xiao et al. [50,118]. Thus, GO and rGO show typical absorption of conductors with zero band gap. The bang gap as commented above is the minimum energy the incident radiation can have for charge separation to create holes at the conduction band. For bare TiO_2_ the band gap oscillates around 3.2–3.3 eV depending on the crystalline phase (anatase or rutile). This energy is too high for the visible light to activate the photocatalytic process. The main role of graphene materials is to reduce the band gap and then make TiO_2_ active under visible light. In addition, due to its high conductivity (i.e. rGO) trapped electrons can be sent away from the TiO_2_ conduction band, thus reducing even further the electron-hole recombination. As examples, Table 4 gives values of band gap and wavelength of radiation that activate the catalyst composite reported in the studies in Table 3. As can be seen, band gap values can be diminished in a significant way so that visible radiation of as much as 689 nm wavelength can activate the catalyst and lead to charge separation. So far, the catalyst prepared by Sheydai et al. [53] has the lowest band gap value, 1.8 eV.

Photoluminiscence (PL) is another technique applied, in this case, to give an estimation of the degree of electron-hole recombination that inhibits the photocatalytic process. This technique was used by Pedrosa et al [49] who reported that the overall PL intensity for their composites significantly diminished when compared to bare TiO_2_. The authors suggested a luminescence quenching, with the carbon phase acting as scavenger of the electrons generated by UV-photo-excitation of TiO_2_. They also detected that the carbon phase induced a blue shift of the highest energy excitation band (360–380 nm), which reveals an exchange of electronic energy between TiO_2_ and the GO derivatives. Xiao et al. [101], on the other hand, observed that the intensity of the emission peak for rGO-TiO_2_ was much lower than that of pristine TiO_2_, which demonstrates that the recombination of photo- generated charges is greatly inhibited by the introduction of rGO.

### 8.2. Doping or Combining Agents in TiO_2_ or ZnO Composites

Different composites have been so far prepared in the works of graphene-based catalysts for photocatalytic ozonation, as observed in Table 3. Only the work of Xiao et al [118] used rGO (0.1 gL^−1^) as a photocatalyst and compared the activity with those of other carbonaceous materials (SWCNTs, MWCNTs, fullerene (C60), and graphitic carbon nitride (g-C_3_N_4_)) and with metal oxides (TiO_2_ and WO_3_). In this study C60, and in particular g-C_3_N_4_, led to the best efficiency results in catalytic ozonation, while rGO as such was not a useful photocatalyst since it is a conductor material rather than a semiconductor. In another study [51], an g-C_3_N_4_-rGO composite was prepared and checked as photocatalyst (0.2 gL^−1^) with better results than with g-C_3_N_4_ alone. In this work, 1% rGO by weight was the optimum concentration. In another three works, the photocatalyst was GO o rGO doped on TiO_2_ at low concentration. Titanium oxide was TiO_2_ P25 in one case [119] or as-prepared TiO_2_ from titanium (IV) butoxide [54] or from (NH_4_)_2_F_6_Ti [52]. Optimum concentrations of GO were 0.33% and 1% (by weight) determined from thermogravimetric analysis for the first two cases, respectively. The authors only gave the optimum amount of GO they used, 0.02 g for the third case. Concentrations of photocatalysts were between 0.25 and 1 gL^−1^. In another two works, the photocatalyst TiO_2_-rGO was also doped with N or S. This was applied in the work of Sheydai et al. [53] who prepared a N-TiO_2_-GO/titan grit sheet with urea as precursor for introducing nitrogen and P25 TiO_2_ for titania. In another work, Pedrosa et al. [49] obtained a series of catalysts and photocatalysts with and without N or S and preparing GO from Hummers and Brodie methods. In this case, they used (NH_4_)_2_F_6_Ti as a titania precursor and urea and benzyldisulfide as N and S precursors, respectively. From TGA they gave carbon percentages in GO and rGO as 2.5–3% and 2%, respectively. Finally, Wu et al. [50] prepared a ZnO-rGO composite from a hydrothermal method, varying the GO percentage from 0.2 to 2% by weight with 0.6% as the optimum one. In this work, the authors used persulfate as oxidizing agent to improve electron capturing. As a summary, it is observed that the percentage of GO on the photocatalyst is always lower than 1% to avoid light shielding on active sites of the semiconductor used.

### 8.3. Reactors and Radiation Sources Used

In most of the works shown in Table 3, a tank operating in semi-batch mode was used. Thus, the suspension (catalyst and organic compound solution) was charged and ozone was continuously bubbled for the reaction time. Agitation was provided magnetically or by bubbling in some cases. The reactor volume oscillated from 7.5 mL [49] to 1 L [51,53]. Regarding the radiation source, both lamps and LEDs were used. Low-, medium-, or high-pressure mercury lamps emitting from the UV-C to visible region are common in these works. Also, Xe lamps simulating sun radiation were applied. In this case, UVA cut-off filters allowed only visible radiation to reach the suspension. In two works [53,54], visible radiation LEDs, mainly emitting at 432 and 425 nm radiation, respectively, were employed to attain a more sustainable environment. Intensity of radiation changed depending on the lamp or LED radiation emitted.

### 8.4. Variables Studied and Experimental Results

As far as the variables studied are concerned, most of the works in Table 3 limit their study to comparing what happens when different ozone processes, adsorption, and photocatalytic oxidation are applied. In these cases, results of compound removal and, in a few cases, TOC removal with time are given. The results of works from Table 3 show that, in all cases, GO-based photocatalytic ozonation is the best process to achieve the highest compound and TOC removal, though in many cases the differences are small compared to those reached with other oxidations. It can also be seen that adsorption or direct photolysis hardly reduces concentration of the organics treated. Differences are negligible or null in some cases, for example when primidone treated with different ozone advanced oxidations included a TiO_2_-GO/O_3_/visible LED process. In some works, an extra oxidizing compound is added such as hydrogen peroxide or persulfate. In both cases, an increase in compound removal is observed. Thus, in the work of Alkandari et al. [119] the addition of hydrogen peroxide to the TiO_2_-GO photocatalytic ozonation process led to a nearly 25% oxidation increase. With this oxidant, Wu et al. [50] observed complete PFOA removal after 4 h reaction, about 20% increase compared to the persulfate free ZnO-rGO photocatalytic ozonation process. While removal of initial organics does not show, as indicated above for many cases, a considerable difference among ozone processes, mineralization, that is, transformation of organic matter (from initial compound and intermediates formed) is significantly different when using photocatalytic ozonation compared to other ozone processes. For instance, in the case of primidone, it was seen [54] that 1% TiO_2_-GO photocatalytic ozonation leads to 82% TOC removal after 2 h, while GO free TiO_2_ photocatalytic ozonation gives 65% TOC reduction. These figures should be highlighted since the radiation source was a set of LEDs emitting radiation at 425 nm, that is, visible light. 

Apart from ozone process comparison, the influence of other variables has also been studied. These variables are catalyst concentration, percentage of GO on the catalyst mass, intensity of radiation applied, concentration of ozone in the feeding gas, pH, etc. One important variable was the percentage of GO doped on TiO_2_. Activity of TiO_2_ or ZnO is highly reduced if GO is present at a percentage higher than 1%. This is confirmed in the works where this variable was verified. For instance, Checa et al. [54] found 0.75% GO as the optimum percentage (in fact, this was 1% as determined by TGA). Low percentages were observed as optimum in the works of Wu et al. [50] with 0.6% rGO or Yin et al. [51] with 1% rGO in their ZnO-rGO and gC_3_N_4_-rGO photocatalysts, respectively. In some others an optimum amount (not a percentage) was given, as in the work of Liao et al. [52] who found 0.02 g rGO as the optimum quantity between 0.01 and 0.03 g in their TiO_2_-rGO photocatalyst. Higher GO or rGO percentages present negative results because the GO sheet may wrap the active photocatalyst particles to decrease the numbers of TiO_2_ active points that radiation can reach. Finally, Sheydai et al. [53] studied the influence of the water matrix by conducting some experiments in well water. They observed, after a 2-h reaction, that cefixime removal dropped from 80% in ultrapure water to 33% in well water as a consequence of natural scavengers that inhibit the hydroxyl radical reaction. These authors extended the oxidation time in well water and after 6 h they reached 55% cefixime removal. In ultrapure water the oxidation does not lead to an increase percentage removal for reaction times higher than 2 h.

### 8.5. Photocatalyst Stability and Activity

Three works from Table 3 have also studied photocatalyst stability and activity by conducting cycles of photocatalytic ozonation runs of fresh compound aqueous solutions with the same photocatalyst that, after each cycle, was separated from water, dried and used again. The numbers of cycles varied from 3 to 5 with a reaction time of 2 h or 45 min. In all cases, only a slight decrease of activity was observed, at less than 5%. Regarding stability, Checa et al. [54] observed no release of TOC from TiO_2_-GO catalyst when it was first ozonated for 1 h. No metal leaching was either observed or reported.

### 8.6. Kinetic and Mechanism Studies

Mechanism and kinetics is undoubtedly the least studied aspect of the works in Table 3. In many cases, the studies are limited to determining the apparent pseudo first order rate constants (or zero order in some cases) of the processes or to observe the effect of the presence of different scavengers to clarify mainly whether oxidation is due to direct ozonation, hydroxyl radical oxidation or to the action of oxidizing holes from the valence band of the semiconductor. For instance, Wu et al. [50] used tBA, methanol, and triethanolamine to scavenge hydroxyl and sulfate radicals and positive holes while studying PFOA/TiO_2_-rGO photocatalytic ozonation. They observed the main participation of these species in the process. Checa et al. [54], used tBA and oxalic acid to check the active presence of hydroxyl radicals and positive holes. In this case, the main species responsible for primidone removal and TOC were hydroxyl radicals. Sheydai et al. [53] used chloroform, EDTA, and 1-butanol as organic scavengers and sulfate, chloride, bicarbonate, and phosphate as inorganic scavengers to check their influence on cefixime removal. All these scavengers act negatively on cefixime removal, so many short-lived species (hydroxyl and superoxide ion radicals, positive holes) play a role in the process. 

Only Sheydai et al. [53] presented a section dedicated to identification of intermediates. In the oxidation of cefixime with GC/MS they identified eight intermediates but they did not provide a possible mechanism of reactions among them. In another work, Xiao et al. [118] calculated what they called a coupling coefficient, that is, the ratio between zero order rate constants of the gC_3_N_4_-rGO photocatalytic ozonation process and that of the sum of the individual processes (catalytic ozonation and photolysis). The authors reported a value of 95, which had never been achieved with any other AOPs they had studied up to then. In the work of Checa et al. [54], both the R_CT_ parameter [89] and ozone consumption per TOC consumed were determined. R_CT_ values clearly show that TiO_2_-GO visible LED photocatalytic ozonation generated a concentration of hydroxyl radicals about 7 and 3 times higher than with ozonation and photolytic ozonation, respectively. On the other hand, ozone consumption per TOC removed was about 3 and 5 times lower than with ozonation and photolytic ozonation, respectively.

Only Wu et al. [50]. suggested a mechanism of reactions involving hydroxyl and sulfate radicals to explain their results on persulfate-aided ZnO-rGO photocatalytic ozonation of PFOA. However, no kinetic equations were derived and verified with this mechanism.

Apart from apparent first- or zero-order kinetics, Sheydai et al. [53] assumed a Langmuir kinetic equation for their study on cefixime removal with N-TiO_2_-GO/Titan grit sheets. Once the kinetics corroborated the experimental results they concluded that both cefixime adsorption and oxidation simultaneously participated in the process. 

## 9. Conclusions

Metal-based catalysts, especially noble metals and transition metal oxides, are catalytically active for ozone decomposition; consequently, catalytic ozonation has been extensively studied to improve water contaminant removal. However, contamination from metal leaching is a real fact due to the strong oxidative capacity of ozone. Looking ahead to environmental sustainable processes, society is demanding the synthesis and application of metal-free catalysts with good results for wastewater treatment. Graphene-based materials could be considered as potential catalysts, though much research is still needed to assess catalytic activity, stability, reusability and scale-up studies. 

Graphene-based materials offer potential advantages in ozonation reactions, as synergistic effects between adsorption, ozonation, and photocatalysis (if radiation is applied) are expected. The degree of this synergism is highly dependent on a number of factors, such as water characteristics (i.e., nature and concentration of pollutants), type of ozone process (e.g., absence or presence of radiation), and, in particular, the nature of the catalyst. Works so far published report different graphene-based catalyst types. In the absence of radiation (the catalytic ozone process) the nature of the catalyst shows high variability from the use of GO or rGO alone and non-metal doping to preparation of metal oxide composites where mainly iron, manganese and titanium oxides are used in combination with GO or rGO. One of the characteristics of these catalysts is the high percentage GO or rGO in the material with the aim of speeding up decomposition of ozone and providing a high surface to favor adsorption of organic pollutants. Catalytic activity is deeply improved in heteroatom doped-graphene materials that have shown extraordinary activity in ozone reactions. With these doped materials catalytic activity has been reported to follow the sequence: N-rGO > P-rGO > B-rGO > rGO > S-rGO. Also, composites of iron and manganese oxides-rGO are the most studied and show high activity and good reusability. Despite providing a high specific surface, little adsorption onto graphene-based materials is observed during catalytic ozonation of water contaminants. Therefore, adsorption contributes to a small extent to the removal of water pollutants by catalytic ozonation. Single ozonation, then, is the benchmark for examining catalytic performance of graphene-based materials. The main ROS species generated in graphene-mediated catalytic ozonation are hydroxyl and superoxide ion radicals and singlet oxygen, although this is still a question of controversy and debate that requires further study. 

In photocatalytic ozonation, GO or rGO are used as dopant agents of semiconductors, mainly TiO_2_. In this process, unlike catalytic ozonation, the presence of GO o rGO is dramatically reduced below 1% to avoid light shielding on active sites of the semiconductor used. In fact, higher GO or rGO percentages present negative results because the GO sheet may wrap the active photocatalyst particles to decrease the numbers of TiO_2_ active points that radiation can reach. However, the PL technique has confirmed an increase in visible light absorption in TiO_2_-GO catalysts and a significant decrease in the band gap (minimum value reached is 1.8 eV compared to that of anatase, at 3.2 eV). This makes the semiconductor active with visible light, which represents a positive step since the visible light of sun radiation speeds up ROS formation. The presence of reduced amounts of GO or rGO helps to prevent electron-hole recombination because of the high conductivity of graphene materials. This is also improved by the presence of ozone that captures electrons given that its high oxidation level yields hydroxyl radicals.

Catalytic ozone processes have been applied to different types of contaminants. Pharmaceuticals are contaminants of special importance due to their potentially hazardous effects (e.g. long-term antibiotic presence in released urban wastewater facilitates adaptation of pathogens, leading to a problem known as antibiotic-resistant bacteria (ARB) or antibiotic-resistant genes (ARG)). In many cases, when comparing single ozonation and graphene-based catalytic ozone processes the so-called synergism effect disappears because ozone direct reactions are the main means of oxidation. However, graphene-based catalytic processes are effectively active for TOC removal, that is, not only initial contaminants are removed but also intermediates and carboxylic acid end products. In this case, synergism is highly important since the catalytic process can lead to 90% or more TOC elimination compared to single ozonation, which barely reaches 30% in some cases.

As a summary, some specific conclusions that can be drawn from this review study concerning advantages of graphene-based materials for the ozonation of water pollutants are the following:

- The type and concentration of defective sites and SOG present in the catalyst highly influence the ozone adsorption and decomposition in ROS. Although the number of SOG depends on the preparation method, epoxides and hydroxyl are the most abundant groups in the basal plane and carboxylic groups in the edges. Also, carbonyl groups can be present, which indicates the existence of defect sizes.
- Graphene-based catalysts can be easily tuned with SOG by employing ozone as a direct oxidant and UV radiation as a reducing agent.
- Metal or metal oxide free graphene-based catalysts are fairly active in the ozonation of water contaminants and can be considered as environmentally-friendly catalysts since their activity does not lead to any metal leaching.
- TiO_2_-GO catalysts present band gaps lower than that of TiO_2_ so that they can absorb visible light. This is another environmental advantage because solar light could be used as radiation source in photocatalytic ozonation. However, it should be taken into account that GO percentages in the catalyst have to be limited to less than 1% to avoid light shielding on active sites.
- Regarding contaminant removal rates, although in some instances ozone alone and both catalytic and photocatalytic ozonation lead to similar individual removal rates, graphene-based catalytic processes significantly enhance the removal of TOC, mostly through generation of HO· and other ROS.

As a final conclusion, it can be said that although graphene-based catalysts of ozone processes have been successfully studied to improve water remediation, these AOPs as emergent technologies have many unknowns that require further research. In particular, synthesis methods to improve reusability, and rigorous kinetics and reaction mechanisms are challenging issues to which the scientific community dedicated to the use of graphene and ozone in water remediation should address their future research.

## Figures and Tables

**Table 1:** List of target pollutants used in ozonation processes catalysed by graphene-based materials. Rate constants of reactions with ozone, k_D_, and hydroxyl radical, k_HO_ *.

Family	Compound	k_D_, M^−1^s^−1 a^	Reference	k_HO_, M^−1^s^−1 b^	Reference
Phenols	Bisphenol A	2.5 × 10^6^	[97]	7.2 × 10^9^	[98]
	*o*-chlorophenol	2.7 × 10^6^	[99]	1.2 × 10^10^	[8]
	*p*-nitrophenol	4.5 × 10^6^	[100]	3.8 × 10^9^	[8]
	Phenol	1.8 × 10^6^	[93]	1.3 × 10^10^	[8]
Pharmaceuticals	Acetylsalicylic acid	N.A.	-	5 × 10^9^	[101]
	Benzotriazole	18.4(pH 2)	[102]	1.7 × 10^10^ (pH = 2)	[102]
		22.0(pH 5)		6.2 × 10^9^ (pH = 10)	
	Diphenhydramine	N.A.	-	5.4 × 10^9^	[103]
	Cefixime	N.A.	-	N.A.	-
	Ibuprofen	9.6	[104]	7.4 × 10^9^	[104]
	Iopromide	<0.8	[104]	3.3 × 10^9^	[104]
	Primidone	3.1	[105]	6.7 × 10^9^	[106]
	Sulfamethoxazole	2.65 × 10^5^ (pH 2)	[107]	8.5 × 10^9^	[108]
		4.15 × 10^5^			
Low molecular weight carboxylic acids	Acetic acid	<5 × 10^−5^	[93]	7.9 × 10^7^	[8]
	Formic acid	100	[93]	3.2 × 10^9^	[8]
	Oxalic acid	<0.04	[93]	7.7 × 10^6^	[8]
Others	Di-*n*-butyl phthalate (DBP)	0.092	[109]	4.64 × 10^9^	[109]
	*N*,*N*-diethyl-m-toluamide (DEET)	0.127	[110]	4.95 × 10^9^	[111]
	*p*-chlorobenzoic acid (p-HBA)	<0.15	[112]	5 × 10^9^	[113]
	Perfluorooctanoic acid (PFOA)	N.A.		<10^5^	[114]

* pH 7 unless indicated. ^a^ Rate constant of ozone direct reaction. ^b^ Rate constant of hydroxyl radical reaction. N.A. Not available.

**Table 2:** Works on graphene-based catalysts used in catalytic ozonation.

Catalysts	Target Pollutants	Reactor	Ozonation Conditions	Process Performance	Reference
GO, oGO, and nOG	4-chlorobenzoic acid (p-CBA) C_0_ = 1 μM	Batch tank reactor. V = 200 mL	Saturated O_3_ stock solution (50–60 mg L^−1^); O_3_ dose = 1 mg L^−1^ pH_0_ = 3–9 C_cat_ = 25 mg L^−1^	p-CBA removal at pH = 9 Ozonation time = 5 min O_3_ alone: 60%; O_3_/nOG: 71%; O_3_/GO: 78%; O_3_/oGO: 87%.	[64]
GO, rGO, and nOG	4-chlorobenzoic acid (p-CBA) C_0_ = 0.5 μM Iopromide (IPM) C_0_ = 1.0 μM	Batch tank reactor. V = 200 mL	Saturated O_3_ stock solution (50–60 mg L^−1^); O_3_ dose = 1 mg L^−1^ pH_0_ = 3–7 C_cat_ = 25 mg L^−1^	IPM removal at pH = 7 Ozonation time = 5 min O_3_ alone: ca. 40%; O_3_/nOG: ca. 40%; O_3_/GO: ca. 90%	[73]
GO, rGO, and g-C_3_N_4_	4-chlorobenzoic acid (p-CBA) Benzotriazole C_0_ = 0.084 M	Batch tank reactor V = 300 mL	C_O3_ = 2 mg L^−1^ pH_0_ = 4.75–6.01 C_cat_ = 0.5 g L^−1^	p-CBA removal at pH = 4.75 Ozonation time = 30 min O_3_ alone: 87%; O_3_/GO: 100%; O_3_/rGO: 100%	[61]
GO	N, N-diethyl-m-toluamide (DEET) C_0_ = 50 μM	Semi-batch reactor V = 250 mL	C_O3g_ = 5 mg L^−1^ F_g_ = 0.4 L·min^−1^ pH_0_ = 2–8 C_cat_ = 20–100 mg L^−1^	DEET removal at pH = 7 Ozonation time = 10 min O_3_ alone: ca. 40%; O_3_/GO: 90–95%	[58]
GO and rGO	p-hydroxyl benzoic acid (p-HBA) C_0_ = 20 mg L^−1^	Semi-batch reactor V = 500 mL	C_O3g_ = 20 mg L^−1^ F_g_ = 0.1 L·min^−1^ pH_0_ = 3.5 C_cat_ = 0.1 gL^−1^	p-HBA removal at pH = 3.5 Ozonation time = 30 min O_3_ alone: ca. 75%; O_3_/rGO: 100%	[59]
GO and rGO	Oxalic acid Acetic acid Formic acid 4-nitrophenol acetylsalicylic acid p-hydroxyl benzoic acid (p-HBA) C_0_ = 20 mg L^−1^	Semi-batch reactor V = 500 mL	C_O3g_ = 50 mg L^−1^ F_g_ = 100 mL min^−1^ pH_0_ = 3, C_cat_ = 0.1 g L^−1^	Oxalic acid removal at pH = 3 Ozonation time = 45 min O_3_ alone: ca. 10%; O_3_/rGO: 90–100%	[60]
GO, rGO, N-rGO, S-rGO, and graphene derivative TiO_2_ composites	Oxalic acid C_0_ = 90 mgL^−1^	Semi-batch tank reactor V = 200 mL	C_O3g_ = 50 mg L^−1^ F_g_ = 150 mL min^−1^ C_O3g_ = 50 mg L^−1^ pH_0_ = 3 C_cat_ = 0.14 g L^−1^	Oxalic acid removal at pH = 3 Ozonation time = 3 h O_3_ alone: ca. 10%; O_3_/GO: ca. 95%; O_3_/rGO: ca. 95%; O_3_/N-rGO: ca. 95–100%; O_3_/S-rGO: ca. 95–100%; O_3_/TiO_2_-GO: ca. 35–60%; O_3_/TiO_2_-rGO: ca. 70–90%	[49]
rGO, N-rGO, and P-rGO	Sulfamethoxazole C_0_ = 50 mg L^−1^	Conical flask V = 100 mL	O_3_ dosage = 2 g h^−1^ F_g_ = 0.4 mL min^−1^ pH_0_ = 5–9 C_cat_ = 1 gL^−1^	TOC removal at pH = 9 Ozonation time = 20 min O_3_ alone: 6%; O_3_/rGO: 17%; O_3_/N-rGO:28%; O_3_/P-rGO: 16%	[67]
rGO and N-rGO	4-nitrophenol C_0_ = 50 mgL^−1^	Semi-batch tank reactor V = 500 mL	C_O3g_ = 50 mg L^−1^ F_g_ = 100 mL min^−1^ pH_0_ = 5 C_cat_ = 0.1 g L^−1^	TOC removal at pH = 5 Ozonation time = 60 min O_3_ alone: ca. 40%; O_3_/rGO: 80–90%; O_3_/N-rGO >95%	[68]
rGO, N-rGO, P-rGO, B-rGO, and S-rGO	4-chlorobenzoic acid (p-CBA) C_0_ = 0.084 M Benzotriazole (BZT) C_0_ = 0.084 M	Batch tank reactor V = 300 mL	C_O3_ = 2 mg L^−1^ pH_0_ = 4.75 (p-CBA) pH_0_ = 6.01 (BZT) C_cat_ = 0.25 g L^−1^	p-CBA removal at pH = 4.75. Ozonation time = 5 min O_3_ alone: ca. 40%; O_3_/rGO: ca. 65%; O_3_/S-rGO: ca. 70%; O_3_/B-rGO: ca. 95%; O_3_/P-rGO >95%; O_3_/N-rGO >95%	[70]
rGO and N-rGO	Oxalic acid C_0_ = 90 mg L^−1^ Phenol C_0_ = 75 mg L^−1^	Semi-batch reactor V = 700 mL	C_O3g_ = 50 mg L^−1^ F_g_ = 100 mL min^−1^ pH_0_ = 3 (oxalic acid) pH_0_ = 8 (phenol) C_cat_ = 0.14 g L^−1^	TOC removal at pH = 8 (phenol oxidation) Ozonation time = 3 h O_3_ alone: ca. 50%; O_3_/rGO: ca. 60%; O_3_/N-rGO: ca. 70–90%	[71]
α-MnO_2_-rGO	Bisphenol A (BPA) C_0_ = 4.4 × 10^−2^ mM	Semi-batch tank reactor V = 200 mL	C_O3g_ = 50 mg L^−1^ F_g_ = 100 mL min^−1^ pH_0_ = 3–10 C_cat_ = 0.1 g L^−1^	BPA removal at pH = 6.25 Ozonation time = 1 h O_3_ alone: 19.1%; O_3_/α-MnO_2_-rGO:90.5%	[45]
γ-MnO_2_-rGO	4-nitrophenol C_0_ = 50 mg L^−1^	Semi-batch tank reactor V = 1 L	C_O3g_ = 50 mg L^−1^ F_g_ = 100 mL min^−1^ pH_0_ = 5 C_cat_ = 0.1 g L^−1^	TOC removal at pH = 5 Ozonation time = 1 h O_3_ alone: ca. 16%; O_3_/γ-MnO_2_-rGO:75–85%	[46]
TiO_2_-GO Fe_3_O_4_-GO TiO_2_-Fe_3_O_4_-GO	Ibuprofen C_0_ = 0.5 µM	Batch tank reactor V = 1L	C_O3_,_0_ = 4 mg·L^−1^ C_cat_ = 30 mg L^−1^	Ibuprofen removal at pH = 7 Ozonation time = 25 min O_3_ alone: 55%; O_3_/GO:76%; O_3_/Fe_3_O_4_-GO:85%	[47]
MnFe_2_O_4_-rGO	Di-n-butyl phthalate (DBP) C_0_ = 0.5 mgL^−1^	Semi-batch tank reactor V = 500 mL	Ozone dosage = 0.4 mg min^−1^ pH_0_ = 7 C_cat_ = 10 mg L^−1^	DBP removal at pH = 7 Ozonation time = 1 h O_3_ alone: 32%; O_3_/MnFe_2_O_4_-rGO:87%	[48]

Nomenclature: C_0_ = initial pollutant concentration; V = working volume; C_O3_: dissolved ozone concentration; C_O3g_: ozone gas concentration; F_g_: gas flow rate; C_cat_: catalyst concentration; pH_0_ = initial pH; GO = graphene oxide; rGO = reduced graphene oxide; nOG = non-oxidized graphene.

**Table 3:** Works on graphene-based catalysts used in photocatalytic ozonation.

Photocatalysts	Target pollutants	Radiation Source and Reactor	Ozonation Conditions	Process Performance	Reference
SWNTs, MWCNTs, C_60_, g-C_3_N_4_, and rGO	Oxalic acid C_0_ = 10^−3^ M	300 W Xe lamp with visible filter Semi-batch tank V = 300 mL	C_O3g_ = 50 mg L^−1^ F_g_ = 100 mL min^−1^ pH_0_ = Not provided C_cat_ = 0.1 g L^−1^	Oxalic acid removal Ozonation time = 30 min O_3_ alone: <5%; O_3_/rGO: ca. 90%; Rad/O_3_/rGO: ca. 95%	[118]
g-C_3_N_4_-rGO (1–4% rGO)	Oxalic acid s C_0_ = 1.1 × 10^−4^ M	500 W High Pressure Xe Lamp Semi-batch tubular reactor V = 1L	m_O3_ = 75 mg h^−1^ F_g_ = 1 L min^−1^ pH_0_ = Not provided C_cat_ = 0.2 g L^−1^	Oxalic acid removal Ozonation time = 40 min O_3_ alone: 7.1%; Vis Rad/O_3_/g-C_3_N_4_-rGO: 70.6%; UV-Vis Rad/O_3_/g-C_3_N_4_-rGO: 93.2%;	[51]
TiO_2_-rGO	Bisphenol A (BPA) C_0_ = 10 mgL^−1^	175 W HighPressure Hg lamp with quartz well (max. at 365 nm) Semi-batch tubular reactor V = 1 L	C_O3g_ = 80 mg L^−1^ F_g_ = 1 mL min^−1^ pH_0_ = Not provided C_cat_ = 0.5 g L^−1^	TOC removal Ozonation time = 45 min O_3_ alone: 18.7%; O_3_/TiO_2_-rGO:19.7%; Rad/O_3_/TiO_2_-rGO: 60.7%	[52]
TiO_2_ (P25)-rGO(0.33% rGO)	Phenol (P), nitrophenol (NP) and chlorophenol (CP) C_0_ = 20 mgL^−1^ Hydrogen peroxide: 70 μL	150W Xe Lamp with visible filter 12 mW cm^−2^ 0.1 L tubular semi-batch reactor	O_3_ from electrochemical generation. H_2_O_2_ was also used. C_O3g_ = 4.1–7.6 g L^−1^ C_H_2_O_2__ = 700 L·L^−1^ when applied C_cat_ = 1 g L^−1^	Compound removals Ozonation time: 30 min O_3_/TiO_2_-rGO: P: 58%; NP: 100%; CP: 90% Ozonation time: 20 min O_3_/H_2_O_2_/TiO_2_-rGO: P: 70%; NP: 100%; CP: 90%	[119]
TiO_2_-N-rGO and TiO_2_-S-rGO (2–3% rGO)	Diphenhydramine (DP) C_0_ = 100 mgL^−1^	Heraeus TQ 150 medium-pressure Hg vapour lamp, 350–700 nm (140 W m^−2^) Semi-batch tank reactor V = 7.5 mL	C_O3g_ = 50 mg L^−1^ F_g_ = 150 mL min^−1^ pH_0_ = Not provided C_cat_ = 1 g L^−1^	DP removal Ozonation time = 1 h O_3_ alone: <5%; Rad/O_3_/TiO_2_-N-rGO: ca. 60%; Rad/O_3_/TiO_2_-S-rGO: ca. 65%	[49]
TiO_2_-N-GO/titan grid sheet	Cefixime, C_0_ = 5–20 mgL^−1^	432 visible LEDs (7.45 W m^−2^) Semi-batch tubular reactor V = 1 L	C_O3g_ = 24 mg L^−1^ F_g_ = 150 mL min^−1^ pH_0_ = 4–10 C_cat_ = 0.25 g L^−1^	Cefixime removal at pH = 10 Ozonation time = 2 h O_3_ alone: ca. 50%; O_3_/TiO_2_-N-GO: ca. 70%; Rad/O_3_/ TiO_2_-N-GO: ca. 80%	[53]
ZnO-rGO	Perfluorocatanoic acid (PFOA) C_0_ = 10 mgL^−1^	254 nm low pressure Hg lamp in a quartz well Semi-batch tubular reactor V = Not provided	Tª = 15–45 °C m_O3g_ = 50 mgh^−1^ F_g_ = 150 mLmin^−1^ pH_0_ = Not provided m_cat_ = 0.2 g C_Persulfate_ = 100 mgL^−1^	PFOA removal Ozonation time = 4 h O_3_ alone: ca. 10%; Rad/O_3_/ZnO-rGO: ca. 85%; Rad/O_3_/ZnO-rGO/S_2_O_8_^2−^: ca. 99%	[50]
TiO_2_-GO (0.3–2% GO)	Primidone (PRM) C_0_ = 20 mgL^−1^	44 visible LEDs (max. 425 nm) 25-455 Wm^−2^ Semi-batch tank V = 0.5 L	C_O3g_ = 10 mgL^−1^ F_g_ = 35 Lh^−1^ pH_0_ = Not provided C_cat_ = 0.125–0.5 gL^−1^	100% PRM removal Ozonation time: 10 min 70% TOC removal Ozonation time: 120 min Optimum GO %: 4	[54]

Nomenclature: C_0_ = initial pollutant concentration; V = working volume; C_O3_: dissolved ozone concentration; C_O3g_: ozone gas concentration; m_O3_ = ozone mass flow rate; F_g_: gas flow rate; C_cat_: catalyst concentration; m_Cat_ = catalyst mass; pH_0_ = initial pH.

**Table 4:** Band gap values of GO-based photocatalysts.

Photocatalyst	Band Gap, eV; (Max. λ, nm)	Reference
TiO_2_	3.31	[118]
rGO	N.D.	
gC_3_N_4_	2.7 (460)	[51]
rGO-gC_3_N_4_	N.D.	
TiO_2_	N.D.	[52]
rGO-TiO_2_	N.D. (415)	
TiO_2_	3.11	[102]
rGO-TiO_2_(P25)	2.96	
TiO_2_	3.2	[49]
GO-TiO_2_	2.9–3.05	
rGO-TiO_2_	3.05–3.1	
TiO_2_ (P25)	3.2	[53]
N-TiO_2_-GO/titan grit sheet	1.8	
rGO-ZnO	N.D.	[50]
TiO_2_	3.02–3.14	[54]
GO-TiO_2_	2.5	

N.D.: no data are given.
